# Use of Treatment Escalation Plans to Guide Care Planning on a Specialist Dementia Unit

**DOI:** 10.1192/bjo.2024.549

**Published:** 2024-08-01

**Authors:** Charlotte Russell, Lucy Calvert

**Affiliations:** 1 NHS Lothian, Edinburgh, United Kingdom; 2 NHS Borders, Borders General Hospital, Melrose, United Kingdom

## Abstract

**Aims:**

This audit reviewed the use of Treatment Escalation Plans (TEPs) on the Borders Specialist Dementia Unit (BSDU). We aimed to use data on completion rates and quality to adapt the TEP form to both improve practice and develop a more specialised form for use in inpatient old age psychiatry.

TEPs improve clinical decision-making in frail and elderly patient populations, and are commonly used on medical wards. However, these forms are primarily orientated towards acute medical environments and may not be appropriate for use in psychiatric inpatient settings, despite the clear benefits they could provide in this patient group.

**Methods:**

This retrospective audit reviewed completion rates and quality of completed TEP forms for 10 BSDU inpatients in December 2023. Data was gathered by reviewing TEPs and using a data collection form to collate information on completion rates and quality of information provided. Both the TEP form and the ReSPECT form were used to review what information would be relevant to include when completing TEP forms for new admissions to BSDU.

**Results:**

Some sections of TEP forms were consistently well-completed – typically those that were quick to complete e.g. tick boxes. However, limitations of the existing TEP form reduced these sections’ usefulness in practice. Most significantly, the form does not indicate whether “ward level care” refers to care on the old age psychiatry ward, or transfer to a medical ward. The “Additional Information” section, which could be used to clarify the patient's ceiling of care and transfer status, was only completed in 40% of cases, despite being particularly relevant to the BSDU patient population. In addition, this audit highlighted that there is no process for reviewing TEPs to ensure they remain appropriate for the patient, which is particularly relevant for old age psychiatry inpatient populations due to their advancing frailty and quickly changing clinical picture.

**Conclusion:**

This audit showed that the current TEP form is not ideally suited to old age psychiatry settings. However, this could be improved with simple adaptations such as distinguishing between psychiatric ward care and medical ward care, and adding a review date to ensure these forms are regularly updated in light of the advancing frailty of old age psychiatry inpatient populations. I would also recommend implementing an initial review of TEP forms shortly after patients are admitted, to ensure the information contained on them is accurate and that they are countersigned by the responsible consultant.

